# Supplementary figures and images for: Assessing Heat–Health Vulnerability Through Temporal, Demographic, and Spatial Lenses: A Time-Stratified Case-Crossover Analysis in New York State
Source: Int J Environ Res Public Health. 2025 Jul 16;22(7):1124. doi: 10.3390/ijerph22071124 (PMC12294469; doi:10.3390/ijerph22071124)

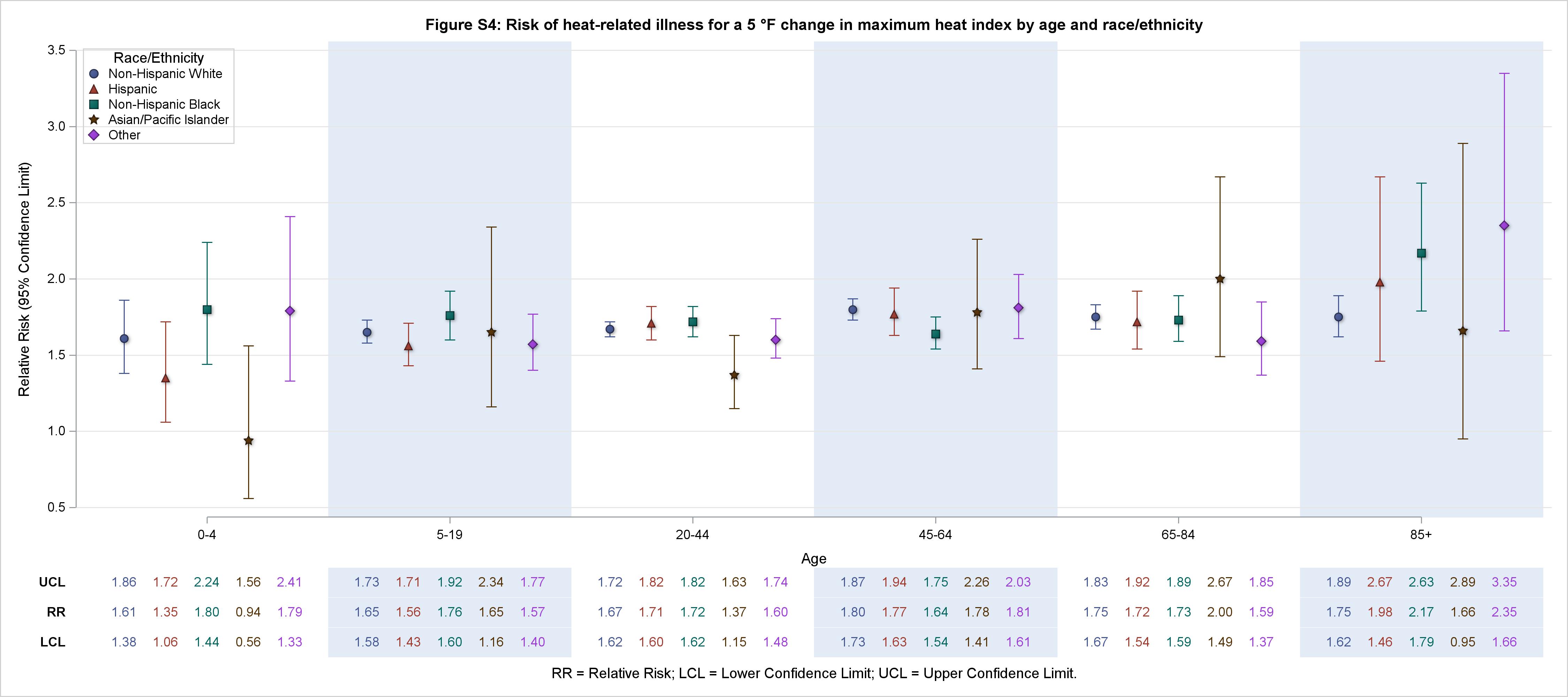

Supplement: Supplementary file 1 [file ijerph-22-01124-s001.zip › Figure S4.jpeg]

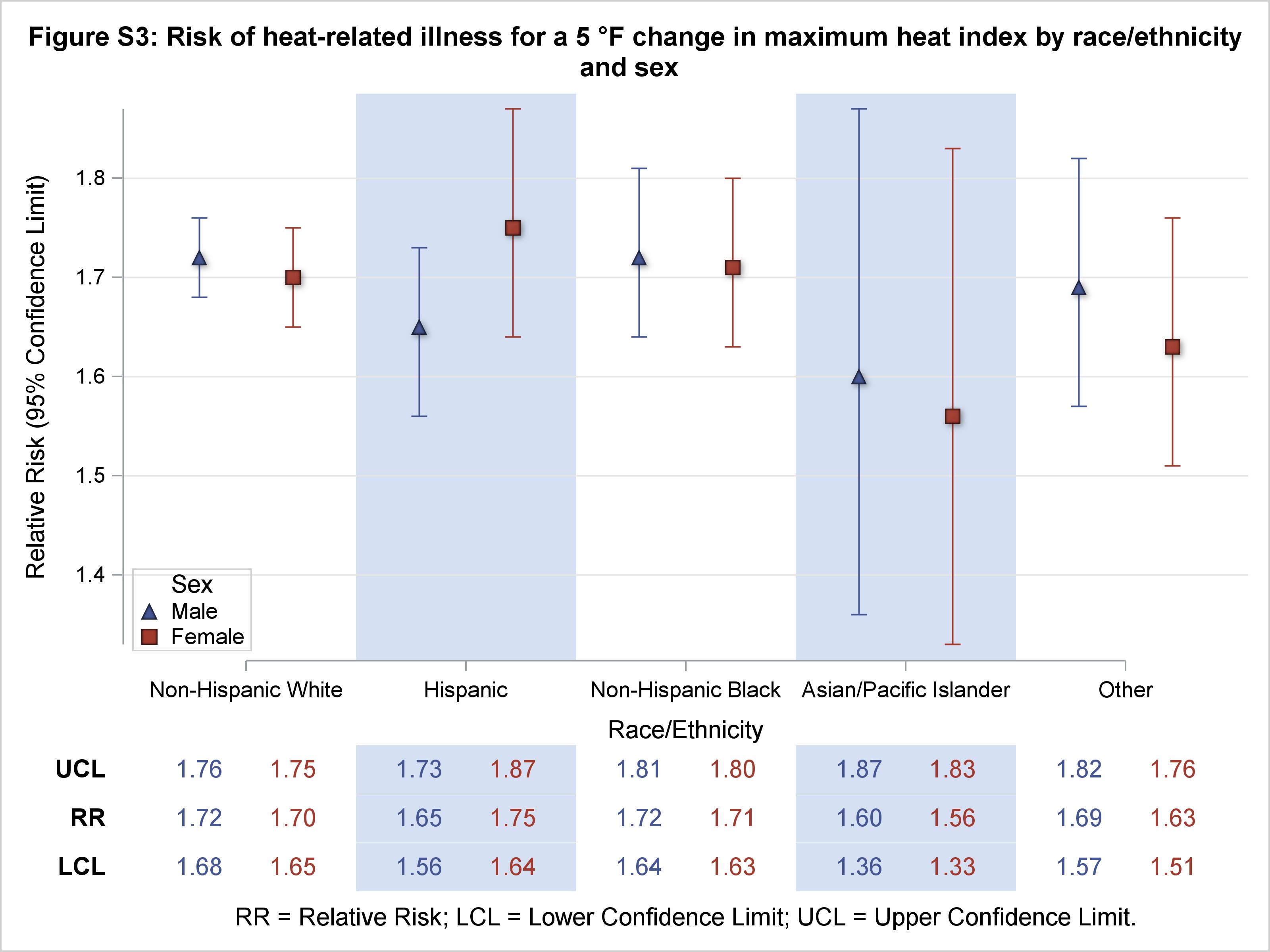

Supplement: Supplementary file 1 [file ijerph-22-01124-s001.zip › Figure S3.jpeg]

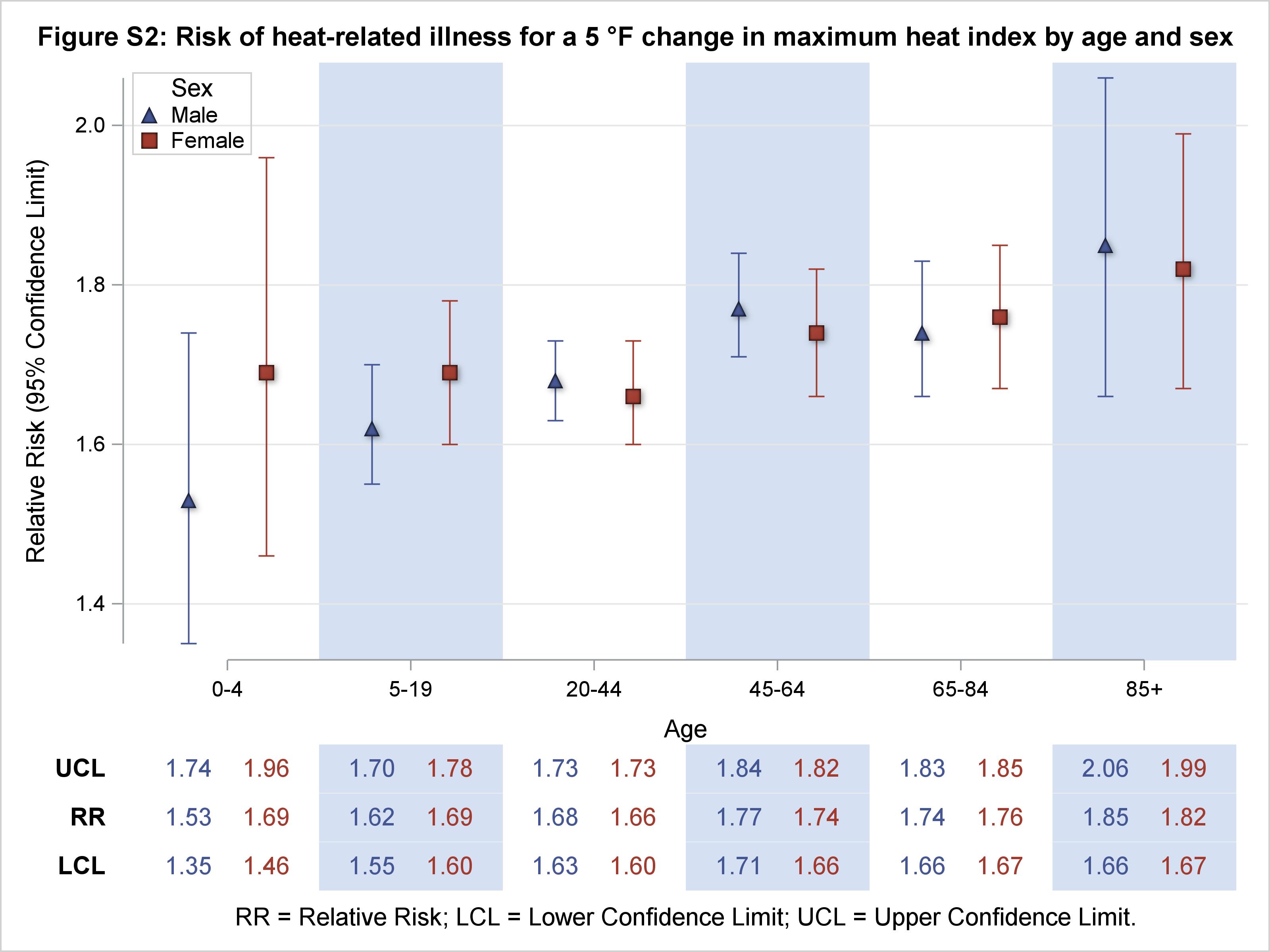

Supplement: Supplementary file 1 [file ijerph-22-01124-s001.zip › Figure S2.jpeg]

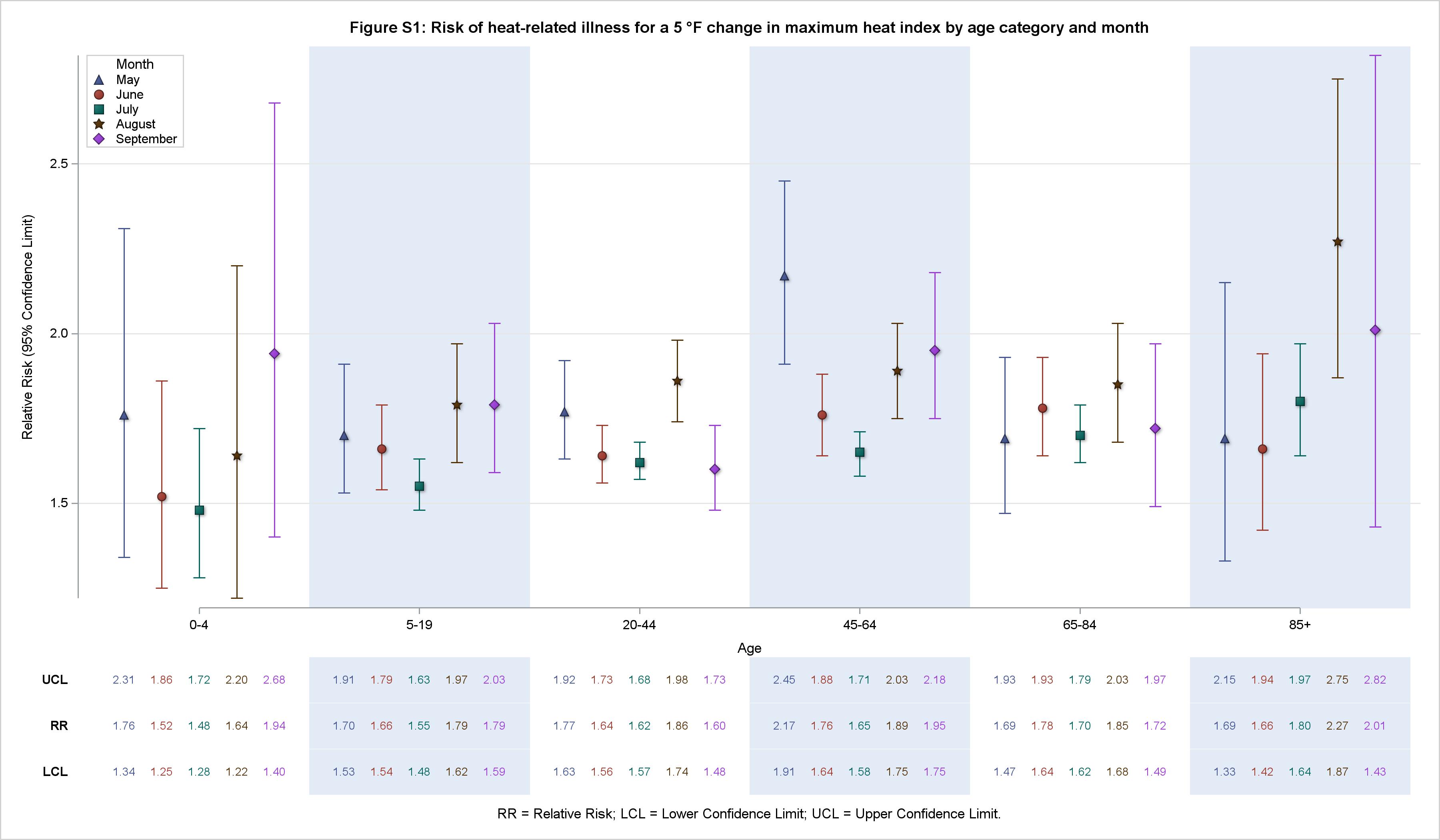

Supplement: Supplementary file 1 [file ijerph-22-01124-s001.zip › Figure S1.jpeg]
